# Influence of Structure-Directing Additives on the Properties of Poly(methylsilsesquioxane) Aerogel-Like Materials

**DOI:** 10.3390/gels5010006

**Published:** 2019-01-28

**Authors:** Marta Ochoa, Alyne Lamy-Mendes, Ana Maia, António Portugal, Luísa Durães

**Affiliations:** 1 CIEPQPF, Department of Chemical Engineering, University of Coimbra, Pólo II, Rua Sílvio Lima, 3030-790 Coimbra, Portugal; marta.ochoa@activeaerogels.com (M.O.); alyne@eq.uc.pt (A.L.-M.); anamaiaf@hotmail.com (A.M.); atp@eq.uc.pt (A.P.); 2 Active Aerogels, Parque Industrial de Taveiro, Lote 8, 3045-508 Coimbra, Portugal

**Keywords:** silica aerogel-like materials, ambient pressure drying, structure-directing additives, glycerol, poly(ethylene glycol)

## Abstract

The effect of glycerol (GLY) and poly(ethylene glycol) (PEG) additives on the properties of silica aerogel-like monoliths obtained from methyltrimethoxysilane (MTMS) precursor was assessed. The tested molar ratios of additive/precursor were from 0 to 0.1 and the lowest bulk densities were obtained with a ratio of 0.025. When a washing step was performed in the sample containing the optimum PEG ratio, the bulk density could be reduced even further. The analysis of the material’s microstructure allowed us to conclude that GLY, if added in an optimum amount, originates a narrower pore size distribution with a higher volume of mesopores and specific surface area. The PEG additive played a binder effect, leading to the filling of micropores and the appearance of large pores (macropores), which caused a reduction in the specific surface area. The reduction of the bulk density and the microstructural changes in the aerogels induced by adding a small amount of these additives confirm the possibility of fine control of properties of these lightweight materials. The achieved high porosity (97%) and low thermal conductivity (~35 mW·m^−1^·K^−1^) makes them suitable to be used as thermal insulators.

## 1. Introduction

Silica aerogels are 3D nanostructured materials obtained by sol-gel technology, using usually tetra-alkylorthosilicates as precursors (TMOS—Si(OCH_3_)_4_ or TEOS—Si(OCH_2_CH_3_)_4_). Their solid skeleton is composed of interlinked structural units of a few nanometers in size and a micro/mesoporous network. These aerogels exhibit unique properties, typically, low apparent density (~100–200 kg·m^−3^), high porosity (>90%), surface area (>500 m^2^·g^−1^) and transparency (~90%) [1,2,3]. Their high porosity results in very low thermal conductivity (tens of mW·m^−1^·K^−1^), sound velocity in the material of ~100 m·s^−1^ and a dielectric constant of ~1.1 [2]. These properties make the silica aerogels suitable for numerous advanced applications. They have already proved superior performance as acoustic insulators, storage and absorbing media, catalysts supports and sensors. However, the area in which silica aerogels are more widespread and effectively used is in thermal insulation for skylights and windows as well as for aerospace applications [1,4].

Nevertheless, the aerogels synthesized from orthosilicates are brittle and absorb moisture due to their hydrophilic character [1], which can modify their insulation capability. To avoid these problems, hydrophobic, flexible and less dense xerogels/aerogels can be obtained using methyltrimethoxysilane (MTMS) as precursor [3,5,6,7,8,9]. The increase in flexibility and decrease in bulk density are mainly due to the macropores originated by the presence of non-reactive methyl groups. However, these larger voids also inhibit the thermal conductivity to decrease down to the typical values of silica aerogels, since the contribution of the gaseous phase for heat transfer increases. The presence of additives during the sol-gel process can alter the final properties of these silica aerogels, including their pore structure. Even though glycerol (GLY) and poly(ethylene glycol) (PEG) are usually added to silica systems as drying control chemical additives (DCCAs), several works already reported their influence in the pore size distribution and in the microstructure [10,11,12,13,14,15,16]. The selection of these two additives for this work was based on their expected effects at these levels, already reported in the literature for silica aerogels/xerogels—Table 1.

Rao and Kulkarni [12] refer that glycerol inhibits the prolongation of the reaction with water and, consequently, the pore size distribution in the final material becomes more narrow and uniform. PEG also has an effect on the pore size, acting as a pore template (porogen), and by varying its amount and molecular weight, it is possible to adjust the pore size and mechanical properties of the final material [2,14]. In low concentrations, it contributes to the decrease in the material’s density and it strengthens the solid network. Moreover, PEG helps the distribution of the silane precursor in the solution. From a deeper analysis of Table 1, it can be concluded that there is an upper limit in the molar amount of GLY (GLY/precursor ≈ 0.8) that can be used if monoliths are required, and also that this additive leads to an increase of the specific surface area/porosity of the aerogels in the studied systems. On the other hand, it appears that PEG induces a decrease in the specific surface area of the produced aerogels/xerogels.

In this work, the influence of GLY and PEG on the properties of silica-based aerogel-like materials obtained with the MTMS precursor and dried by APD is assessed, which was never reported in the open literature. The tested molar ratios of additive/MTMS are in the range 0–0.1. Special attention was given on the possibility of obtaining less dense aerogels with these additives, thus with properties closer to the supercritically dried ones. The final materials were characterized at chemical, physical and structural levels.

## 2. Results and Discussion

The aerogel-like materials were obtained by a two-step acid-base catalyzed sol-gel process as already reported by Durães et al. [9], and following the methodology developed by Rao and co-workers [3], but now adding GLY or PEG along with the silica precursor. The synthesized silica materials are denoted as S-(PEG or GLY)-M, where M is the additive/MTMS molar ratio. The sample with the washing step was named S-PEG-0.025_W. It is worth mentioning that one sample was obtained without additive (see Table 2), for reference, being named S-0.

The bulk density was selected as a key indicator, thus the obtained results in this work were compared with those achieved in an earlier work published by our research group [15], for a silica aerogel synthesized from MTMS in the same sol-gel conditions and one MTMS-derived silica aerogel containing PEG (molar ratio PEG/MTMS of 0.01), both dried with a continuous flow of supercritical CO_2_. The bulk density achieved in the first was 48.8 ± 1.9 kg·m^−3^ and for the sample with PEG was 41.7 ± 1.1 kg·m^−3^ [15]. The densities (bulk and skeletal) and porosities obtained for the aerogel-like materials produced in this work are presented in Table 2. The bulk density exhibits a minimum for an additive/MTMS molar ratio of 0.025, being this minimum coincident for both additives (72.7 ± 2.3 kg·m^−3^ for PEG and 76.1 ± 3.3 kg·m^−3^ for GLY). The decrease verified in the bulk density at the minimum point relatively to the sample without additive is more significant with PEG. This system is also the one in which the bulk density increases more significantly with the increase of additive after the optimum molar ratio.

**Figure 1:**
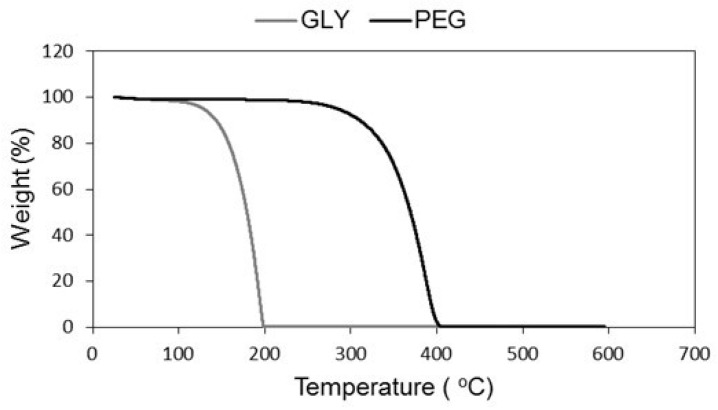
Thermogravimetric analysis of the additives glycerol (GLY) and poly(ethylene glycol) (PEG).

The observed densities can be obviously affected by the amount of additive retained in the final material, so an extra step of washing was made, before drying, in the sample containing a PEG:MTMS molar ratio of 0.025, which was the sample that presented the lowest bulk density value. After this procedure, an even greater reduction was verified, with the density achieving a value of 46.1 ± 3.8 kg·m^−3^, being this a much more similar result to the one obtained for the supercritically dried aerogel with PEG (41.7 ± 1.1 kg·m^−3^ [15]). This variation can be attributed to the PEG removal during the methanol washing step, since the achieved temperatures during the drying step are not high enough for thermal degradation of PEG chains, as showed by the thermogravimetric analysis performed for both additives, Figure 1.

During drying, a step with a temperature of 200 °C was applied, this value being superior to the required temperature for GLY decomposition (onset temperature = 165.0 °C, end temperature = 198.2 °C, Figure 1). This result is in agreement with other works found in the literature, in which glycerol presents a single fast weight loss step around 200 °C [21,22]. However, the drying temperature is not enough for PEG removal, since the onset temperature for thermal degradation of this additive is 342.9 °C and the end of the phenomenon is at 398.8 °C (Figure 1). Considering these data, it can be concluded that there is a high probability of some amount of PEG retained in the final material after the drying stage. Therefore, the extra washing step can help to reduce the PEG amount in the final material. Another possibility to assure the removal of PEG would be to increase the drying temperatures over 400 °C. However, based on the results of Afonso et al. [23], MTMS-based xerogels treated at 300 °C showed a loss of ~10% of their initial weight, and at a temperature of 450 °C the methyl groups were in part oxidized, the hydrophobic nature was lost and a sharp density increase was observed. Thus, this thermal treatment is not favorable for the properties of MTMS-based aerogels/xerogels.

Based on the results of Table 2, the major part of the remaining discussion will focus only on the reference sample (S-0), two samples of each set (S-GLY-0.025, S-GLY-0.1 and S-PEG-0.025, S-PEG-0.1), plus the washed sample for the PEG system (S-PEG-0.025_W). This selection will allow the comparison of the results for the interval extremes of the additives amount with the point where the minimum bulk density was achieved.

In terms of monolithicity of the synthesized materials, all samples of the GLY set were cylindrical monoliths and exhibited white color. For the PEG additive, monoliths were obtained only for samples with a molar ratio additive/MTMS up to 0.075 (included). The sample with the highest amount of PEG (S-PEG-0.1) was cracked, thus PEG/MTMS = 0.075 can be considered the molar ratio limit for obtaining monolithic materials, considering the range and conditions studied in this work. With the increasing amount of PEG, the sample’s surface turned from white to a light yellowish color, while the interior remained white. This is due to the accumulation of PEG at the material’s surface in contact with the test tube during drying.

**Figure 2:**
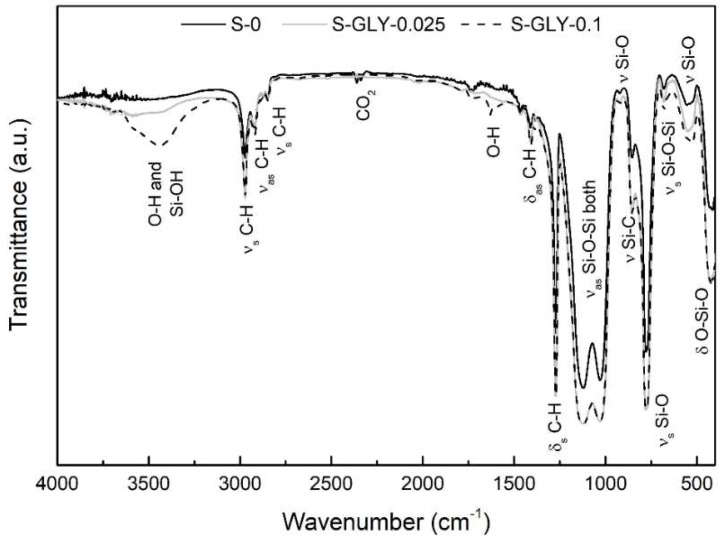
FTIR spectra for aerogel-like samples with and without GLY additive. ν—stretching vibration; ν_s_—symmetric stretching vibration; ν_as_—asymmetric stretching vibration; δ—deformation vibration; δ_s_—symmetric deformation vibration (bending); δ_as_—asymmetric deformation vibration (bending).

**Figure 3:**
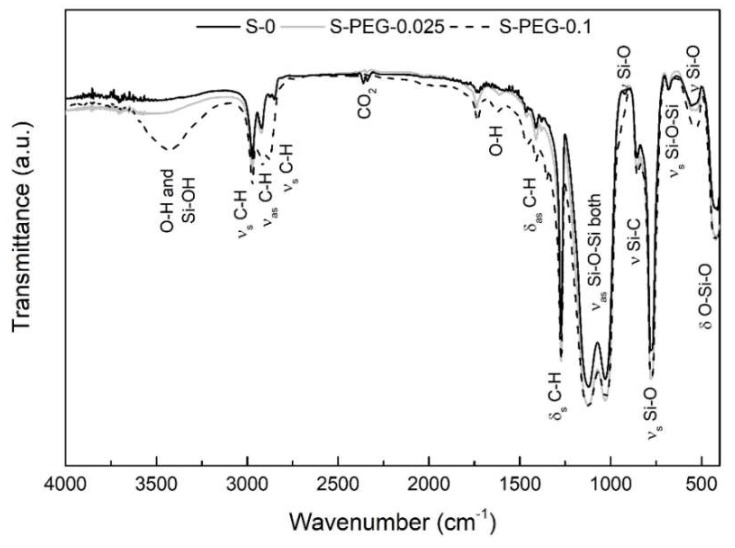
FTIR spectra obtained for aerogel-like samples with and without PEG additive. ν—stretching vibration; ν_s_—symmetric stretching vibration; ν_as_—asymmetric stretching vibration; δ—deformation vibration; δ_s_—symmetric deformation vibration (bending); δ_as_—asymmetric deformation vibration (bending).

Figure 2 and Figure 3 present the FTIR spectra obtained for aerogel-like materials without and with additives, in order to make a comparison of their chemical structure. The assignment of the peaks of the spectra was based on literature FTIR data for silica based systems [24], for general organic bonds [25] and for environmental constituents [26]. All the FTIR spectra are very similar in what concerns the existent types of bonds. The vibration bands found in the samples agree with the expected chemical bonds in these silica-based materials, which have a Si–O–Si (silica) solid network with a methyl group per silicon and hydroxyl groups at the network ends. For the sample S-GLY-0.1 (Figure 2), a higher amount of O–H contribution between 3000 and 3600 cm^−1^ is noticeable when comparing with the samples S-0 and S-GLY-0.025. One possible justification for this behavior is related with the retention of a small amount of GLY in the material structure during drying, because this additive has three OH groups per molecule (HOCH_2_–CHOH–CH_2_OH).

The spectrum of sample S-PEG-0.1 (Figure 3) shows more intense absorptions in the regions related with C–H bonds (1350–1400 cm^−1^; 2800–3000 cm^−1^) and O–H bonds (near 1600 cm^−1^ and 3000–3600 cm^−1^). With the increase of PEG amount, a slight enlargement in the bands near the region of 1200 cm^−1^ is observed. This modification happens due to the overlap of the strong absorption band of aliphatic ethers (–CH_2_–O–CH_2_–), that occurs between 1150–1020 cm^−1^, with the two intense silicon–oxygen bond vibrations that appear mainly in the 1200–1000 cm^−1^ range. Thus, as the amount of PEG increases, a larger band is observed in the infrared spectrum, what can be explained by the presence of the PEG additive (H[OCH_2_CH_2_]*_n_*OH) in the aerogel-like structure, due to the non-release of part of this additive during drying.

It can also be noted that absorptions correspondent to Si–O bonds are generally more intense in the samples with additives when compared to sample S-0. Considering this observation, it can be concluded that these additives appear to favor the condensation reactions, resulting in a slight increase of the condensation extent.

**Figure 4:**
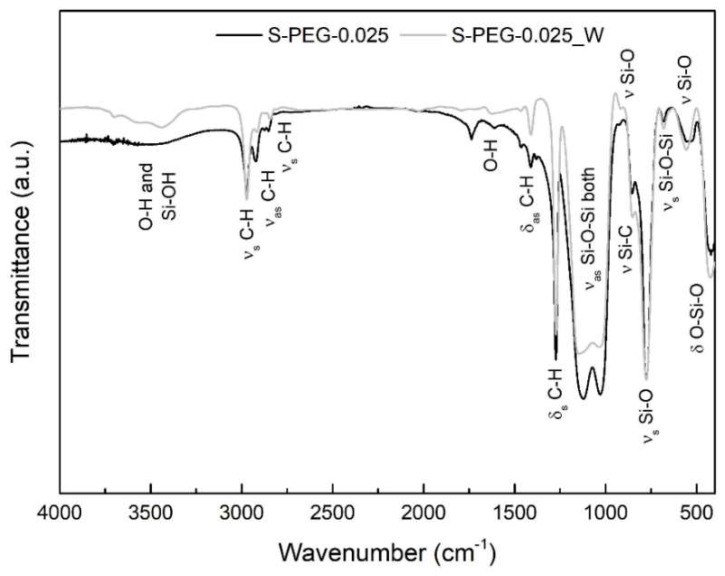
FTIR spectra obtained for the aerogel-like materials containing a PEG/Si molar ratio of 0.025, with and without the washing step. ν—stretching vibration; ν_s_—symmetric stretching vibration; ν_as_—asymmetric stretching vibration; δ—deformation vibration; δ_s_—symmetric deformation vibration (bending); δ_as_—asymmetric deformation vibration (bending).

In order to confirm the influence of the washing step, FTIR was also performed for the sample with the methanol washing (S-PEG-0.025_W) and the obtained results were compared with the sample with the same amount of PEG without washing (S-PEG-0.025), as shown in Figure 4. The spectra are very similar in terms of the types of bonds, indicating that the washing step with methanol does not degrade the chemical structure of the aerogel-like material.

**Figure 5:**
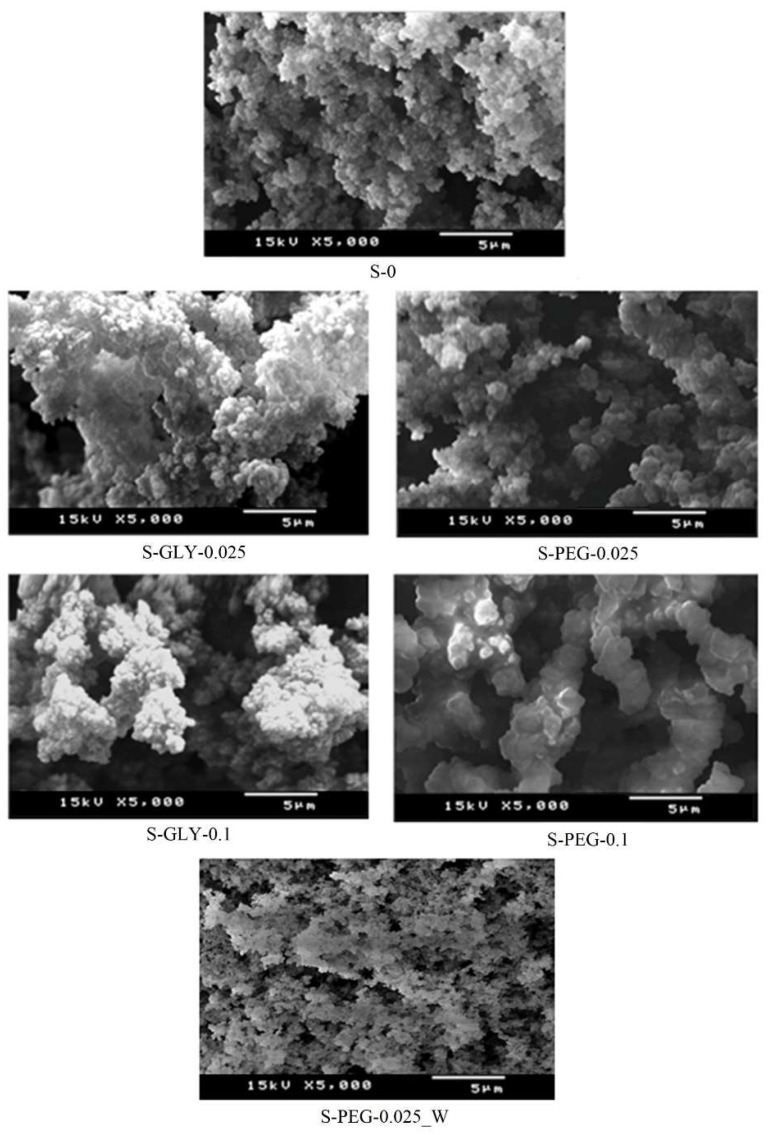
SEM images of the typical microstructures of the aerogel-like materials synthesized with and without additives.

SEM micrographs of Figure 5 show the typical microstructures of the obtained aerogel-like materials. The sample without additive exhibits a foam-like structure, composed by very small interlinked structural units with size much smaller than 1 μm and pores sizes mainly in the range of mesopores. The aerogel-like materials with additive/MTMS molar ratio of 0.025 already show some differences from the non-additivated sample. In the case of the S-GLY-0.025 sample, the structural units of the material seem to be more agglomerated when compared to the structure of S-0, showing a more closed structure in the agglomerates but with larger pores (macropores) between the agglomerates. The structure observed in the S-GLY-0.1 sample is very similar to that of S-GLY-0.025 and no further conclusions can be drawn from the comparison of SEM images of these two samples.

For the S-PEG-0.025 sample, the structural units and the voids appear to be larger than in S-0. This can be due to an enhancement of condensation by the presence of PEG, but more certainly due to the filling of pores with PEG (binder effect). As already mentioned, this additive may have been partially retained in the structure due to its high boiling point. The sample S-PEG-0.1 shows a more significant binder effect of PEG on the structural units, greatly reducing the number of pores and increasing the size of the existing pores. When analyzing the sample S-PEG-0.025_W, it is possible to observe a structure much more similar with S-0 than with S-PEG-0.025, presenting small interlinked structural units. This, once again, indicates the removal of PEG during the washing step.

**Figure 6:**
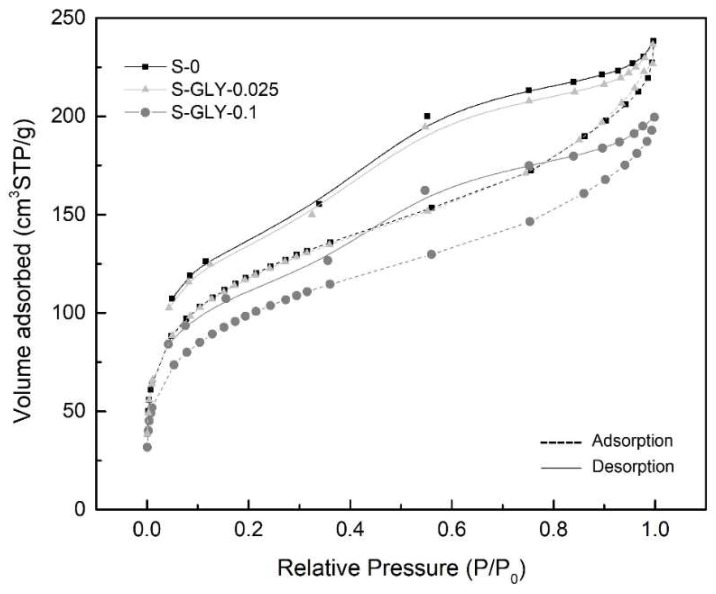
Nitrogen gas adsorption/desorption isotherms for aerogel-like materials with and without GLY additive.

**Figure 7:**
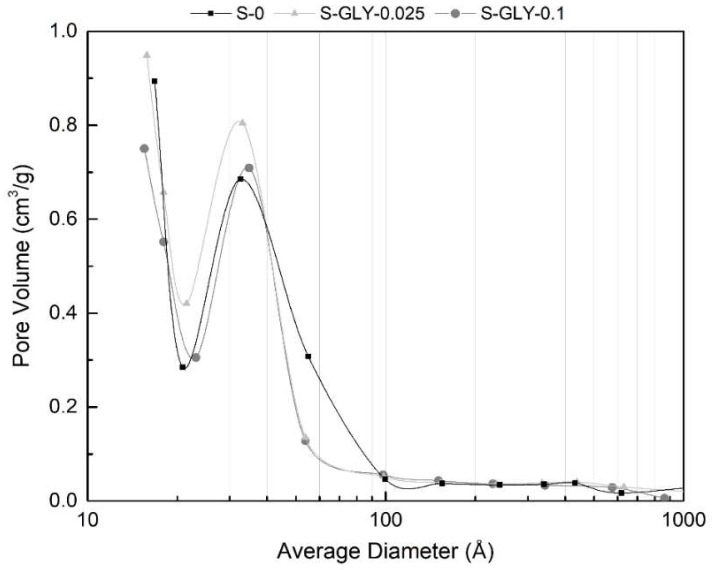
Pore size distributions of aerogel-like materials with and without GLY additive.

**Figure 8:**
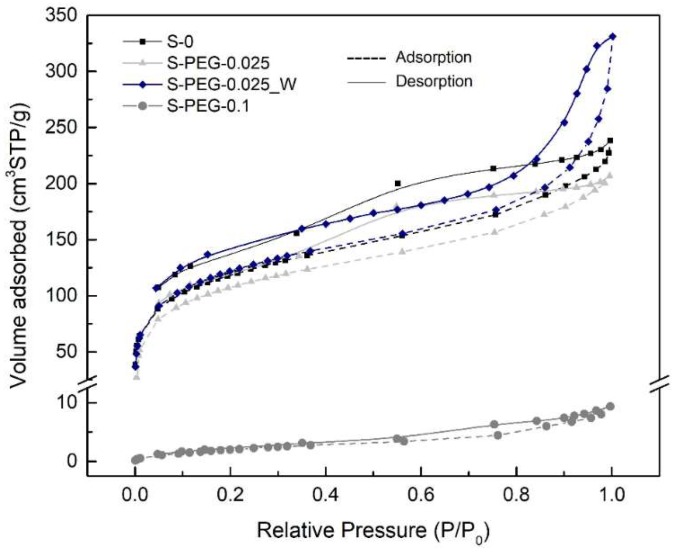
Nitrogen gas adsorption/desorption isotherms for aerogel-like materials with and without PEG additive.

**Figure 9:**
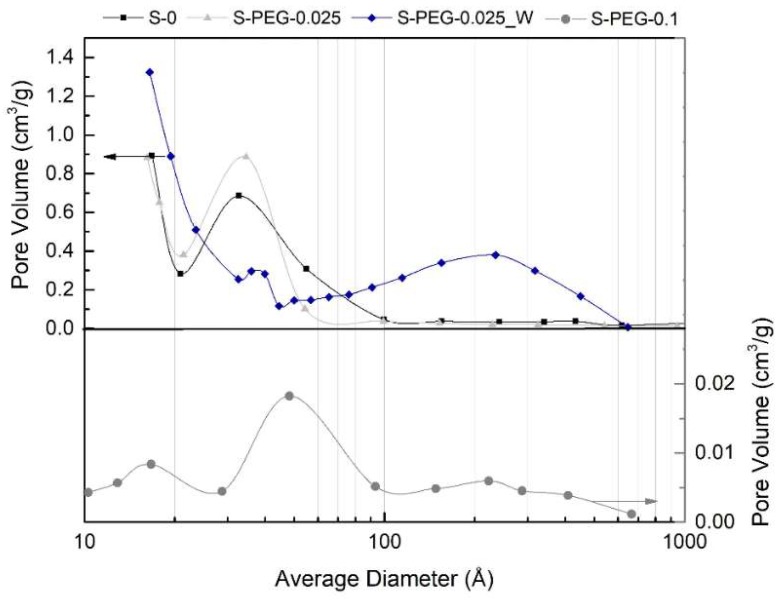
Pore size distributions of aerogel-like materials with and without PEG additive.

Further information on the pore structure was obtained by the nitrogen gas adsorption technique. The results of specific surface areas, pore volumes and sizes are given in Table 3, and the registered isotherms and pore size distributions are presented in Figure 6, Figure 7, Figure 8 and Figure 9. With the exception of sample S-PEG-0.1, the values obtained for the specific surface area are in agreement with the results found in the literature for different MTMS-based systems [27,28]. While for the measurement of the specific surface area all pores are covered by adsorbed nitrogen (low relative pressure), only mesopores are entirely and accurately quantified in terms of pore volume (*V*_P_), causing a deviation in the average pore size value [29]. This technique is demanding and presents some limitations regarding the characterization of the pore structure of aerogels [30,31]. Therefore, the calculated average pore sizes featured in Table 3 were obtained both by the BJH desorption model and by the equation 4*V*_P_/*S*_BET_, where the pore volume was assessed using bulk and skeletal densities [32]. For all the materials, as expected, the values of calculated pore volume from densities are superior than the ones determined by the BJH desorption method, since the first approach takes into account all pore sizes and not only the mesopores.

The obtained isotherms are of type IV for all the samples, with the characteristic hysteresis loop between adsorption and desorption branches that indicates a mesoporous structure in the materials. Considering the isotherm profiles, it is clear that the adsorbed volume of nitrogen in the materials with GLY (Figure 6) increases slightly and then decreases, when the GLY/MTMS molar ratio increases from 0 to 0.025 and from 0.025 to 0.1, respectively. The specific surface areas and the pore volumes of the materials must follow the observed trends of the adsorbed volume, which is confirmed in Table 3.

Therefore, it can be concluded that the addition of a very low amount of GLY (GLY/MTMS = 0.025) leads to an increase of the specific surface area and a small increase of the pore volume, with a consequent slight decrease in the bulk density (Table 2). However, a higher amount of GLY (GLY/MTMS = 0.1) leads to the decrease of the surface area and pore volume, which is in agreement with the increase in the bulk density.

An explanation for the effect of this additive on the pore volume and surface area can be found in the pore size distribution curve obtained by the BJH desorption method (Figure 7). It was already mentioned in the introduction that glycerol would lead to a narrowing of the pore size distribution, which is in fact observed for the mesopores region in Figure 7. In addition to this effect, it is also noted that the sample S-GLY-0.025 shows a higher volume of mesopores between 20 and 40 Å than the others, and the maximum of the distribution curve is shifted to lower pore diameters. This leads to a decrease in the average pore size, if we consider only the mesopores (BJH-desorption average pore size), as can be seen in Table 3. However, if the regions of micro- and macropores are also taken into account (pore size obtained from 4*V*_P_/*S*_BET_), the average pore size of all the samples containing GLY is similar (Table 3).

For the case of PEG (Figure 8), the adsorbed volume of nitrogen always decreases with the increasing of the PEG/MTMS molar ratio, making this decrease very marked from the sample S-PEG-0.025 to the sample S-PEG-0.1. In fact, the S-PEG-0.1 sample presents a very low capability to adsorb nitrogen.

The PEG additive acts as a pore template, causing a variation in the pore size that depends on its molecular weight and amount (Introduction). The filling of pores by PEG leads to an expected decrease in the pore volume and surface area, if PEG is not all released during drying. This effect is seen in this work especially for the S-PEG-0.1 sample, where the filling of smaller pores by PEG resulted in a dramatic decrease in the surface area and pore volume and in an increase of the average pore size (Table 3). This is also observed in Figure 8 by the very low value of the ordinate for this sample (S-PEG-0.1). The modification in the pore size is confirmed for S-PEG-0.1 in Figure 9, where it is possible to see the attenuation of the volume of micropores (<20 Å) and the shift of the mesopores to higher pore diameters. That increase in the pore size is even more remarkable when analyzing the values obtained by the calculation 4*V*_P_/*S*_BET_, with S-PEG-0.1 showing an average pore size in the order of micrometers. The presence of these larger pores is effectively observed by SEM (Figure 5).

From sample S-0 to sample S-PEG-0.025, it is possible to observe a decrease in the specific surface area (not so large as for S-PEG-0.1, Table 3), but in this case there is a small decrease also in the bulk density (Table 2). One explanation for this trend, is the appearance of large macropores (*cf*. Figure 5), which are not detectable by the nitrogen gas adsorption technique (Figure 9) but are confirmed by the small rise in the calculated average pore size (Table 3). The decrease in the specific surface area and the increase in the pore size due to the addition of PEG to silica gels were already mentioned in the literature [10,16], as indicated in Table 1.

When comparing the results of samples S-PEG-0.025_W and S-PEG-0.025, an increase in the specific surface area is verified, which can, once again, indicate the removal of PEG during the washing step. The surface area and calculated pore volume values for the washed sample are even higher than those presented by the sample without additive. The S-PEG-0.025_W also exhibits the lowest value of bulk density among all the analyzed samples. This low value can be justified by the appearance of large mesopores and macropores.

Table 4 shows the contact angles and the thermal conductivities for the aerogel-like materials with and without additives. It can be concluded that the obtained contact angles are all very high, thus the materials are highly hydrophobic. The GLY additive leads to a gradual decrease of the contact angle values. For PEG samples, the contact angle first increases (from S-0 to S-PEG-0.025 and S-PEG-0.025_W) and then decreases (from S-PEG-0.025 to S-PEG-0.075). These observed variations in contact angle can be in part explained by the retention of some amount of the additive in the aerogel-like structure but the surface roughness also affects the obtained values in a more unpredictable way. The more hydrophobic sample is S-PEG-0.025, although it has also the larger uncertainty.

The thermal conductivity values should be inversely proportional to the void’s volume in the sample (thus, proportional to the bulk density), but are also affected by the presence of additives in the aerogel-like structure, especially in the case of PEG (see Table 4). It can be observed that this property is not very sensitive to small variations of the bulk density (Table 2), being the registered values between ~35 and 45 mW·m^−1^·K^−1^. It is verified that the washing step improved the obtained results, reducing the values of thermal conductivity obtained for the PEG molar ratio of 0.025. The obtained value for the washed sample is even lower than the one obtained for a similar system but dried in supercritical conditions (36 mW·m^−1^·K^−1^ at 20 °C) [15] and, for example, silica-cellulose aerogels (39–41 mW·m^−1^·K^−1^) [33]. Moreover, it is has also a competitive performance when compared to the materials conventionally used as thermal insulators, such as cotton (40 mW·m^−1^·K^−1^), wool (30–40 mW·m^−1^·K^−1^), felt (60 mW·m^−1^·K^−1^) and insulation boards (35–160 mW·m^−1^·K^−1^) [34].

These results, combined with the higher values obtained for the material with a PEG molar ratio of 0.075, indicates that the remaining presence of PEG in the material acts negatively in the thermal properties. Even though these are already low values, it is worth to note that these measurements were performed at atmospheric pressure and the thermal conductivity decreases in vacuum condition, the latter positively affecting the insulation performance of these materials in space.

## 3. Conclusions

Silica based materials were synthesized by sol-gel technology using methyltrimethoxysilane as precursor and glycerol and poly(ethylene glycol) as structure-directing additives. The drying of the gels was performed at ambient pressure, producing monolithic aerogel-like materials. The effect of the referred additives on the physico-chemical and microstructural properties of the obtained samples was evaluated, in order to investigate if the use of these additives can help for obtaining materials more suitable for thermal insulation in space (low density and thermal conductivity) using ambient pressure drying. All aerogel-like materials with GLY were monolithic and retained the characteristic white color of the material without additive. On the other hand, for PEG additive, the aerogel-like materials presented a yellowish tone on their surface, more perceptible with higher concentration of the additive, and their monolithicity was lost for a PEG/MTMS molar ratio of 0.1. The yellowish color is due to the retention of a non-negligible amount of the PEG additive in the aerogel-like structure, as confirmed by FTIR results. A minimum value of the bulk density was achieved with an additive/precursor molar ratio of 0.025 for both additives. In the case of PEG, an extra sample with this molar ratio has undergone a washing step before drying, in order to remove the retained additive.

The effect of GLY on the control of pore size distribution is optimum (narrowed distribution with a higher volume of mesopores) with the abovementioned molar ratio, thus the specific surface area increases when this ratio increases from 0 to 0.025 and decreases for higher concentrations of additive. For the materials prepared with PEG additive, the specific surface area always decreased with the increase of the additive concentration due to the filling of smaller pores by PEG. However, when the washing step is performed, PEG is removed from the sample, which leads to an increase in the specific surface area and a reduction in the bulk density. In addition, this sample showed the best properties, with high porosity (~97%), very high hydrophobicity (>146°), relatively low thermal conductivity (~35 mW·m^−1^·K^−1^), making this material more suitable for insulation purposes (thermal, electrical or acoustic) in space than the sample prepared from MTMS without additives.

## 4. Materials and Methods 

### 4.1. Materials

MTMS precursor (CH_3_(OCH_3_)_3_, 98%), methanol (CH_3_OH, 99.8%), GLY (HOCH_2_CH(OH)CH_2_OH, 99%) and PEG (H(OCH_2_CH_2_)*_n_*OH, average MW 600) were used as received. The oxalic acid (C_2_H_2_O_4_, 99%) and ammonium hydroxide (NH_4_OH, 25% in water) catalysts were used in the form of aqueous solutions, prepared with deionized water of high purity (from Millipore ultrapure water system, Burlington, MA, USA).

### 4.2. Synthesis of Silica Aerogel-Like Materials

MTMS is diluted in methanol followed by the addition of an oxalic acid aqueous solution (0.01 M), which favors the hydrolysis of the precursor, forming silanol species (monomers). The additives were added along with the precursor, according to the molar ratios presented in Table 2. After 24 h of hydrolysis, the aqueous ammonium solution (10 M) was slowly added to raise the pH and therefore increase the rate of the condensation reaction between silanols. The solutions have a final volume of approximately 50 mL. These two steps were performed under a controlled temperature of 25 °C. The gelation of the obtained sol occurred inside an oven at 27 °C and subsequently the gels were aged for two days at the same temperature. Finally, the gels were dried at ambient pressure using several temperature cycles: 24 h at 60 °C, followed by three steps, at 100, 150 and 200 °C, of 1 h each. One additional sample with optimum PEG amount was prepared, implementing a washing stage with methanol for four days before the drying.

### 4.3. Characterization

Thermal gravimetric analysis (TGA) of the additives was performed using a TGA-Q500 instrument, from TA Instruments, New Castle, DE, USA. The samples were placed in an alumina pan and heated from room temperature to 600 °C, under a nitrogen atmosphere, with a heating rate of 10 °C·min^−1^. The bulk density of the aerogel-like materials was determined through the mass/volume ratio, by weighting and measuring regular sample pieces. He pycnometry (Accupyc 1330, Micromeritics, Norcross, GA, USA) was used to measure the skeletal density, being the porosity then evaluated using the bulk and skeletal densities. The chemical structures of the materials were assessed by FTIR (FT/IR-4200 spectrometer, Jasco, Easton, MD, USA) using the KBr pellet method. The pellets were prepared with 78–80 mg of KBr and 0.2–0.3 mg of each sample. Wavenumber ranged from 4000 to 400 cm^−1^ with a resolution of 4 cm^−1^. The specific surface area and the pore size distribution of the aerogel-like materials were determined by nitrogen gas adsorption/desorption with a Micromeritics ASAP 2000 analyzer. Before the analysis, the samples were outgassed at 60 °C in vacuum for 24 h. Volumes of the adsorbed nitrogen at five different relative pressures (0.05 to 0.2) were taken at 77 K, in order to obtain the specific surface area by the BET theory. For all BET surface area (*S*_BET_) results, the fitting parameters were: correlation coefficient (*R*^2^) of at least 0.999 and a constant *C* higher than 15. The desorption isotherm and the BJH theory were used for the porosimetry evaluation. The average pore size was also obtained by the simple rule 4*V*_P_/*S*_BET_, where the pore volume (*V*_P_) was obtained using both bulk and skeletal densities (*V*_P_ = [(1/bulk density) − (1/skeletal density)]). The microstructure of the aerogel-like materials was observed by scanning electron microscopy—SEM (JMS-5310, JOEL, Peabody, MA, USA). Finally, the hydrophobicity was obtained via contact angle measurement (OCA 20, Dataphysics, Filderstadt, Germany), using the sessile drop technique with high purity water as test liquid, and the Thermal Constants Analyzer TPS 2500 S, from Hot Disk (Göteborg, Sweden), was used to obtain their thermal conductivities, being the analyses by both techniques performed at room temperature (20–23 °C).

## Figures and Tables

**Table 1:** Summary of evolution of works related with the study of the effect of GLY and PEG in silica aerogels/xerogels.

Chemical System ^a,b^					Drying Method ^c^	Thermal Post-Treatments	Main Conclusions ^d^	Ref.
Precursor(s)	Solvent	Water	Catalyst	Additive(s)				
TEOS 5 g	-	30 g	HCl pH = 1.8 NH_4_OH pH = 8.2	PEG (up to 37.5 wt%) Other: PVA, PAA, PEI, proteins	APD	500–800 °C	- PEG had little influence on silica particle growth of the sol and led to a decrease of the specific surface area of the final material; - PEG influenced the (meso)pores size distribution.	[10] (1990)
TMOS or TEOS 1	-	4–10	HNO_3_ pH = 1.5	GLY or PEG 1 to 5 Other: FA	APD	300 °C 400 °C 500–1100 °C	- PEG and GLY lead to a substantial increase of gelation time and with these additives the produced materials were not monolithic.	[17] (1991)
TMOS 1	MeOH 6–12	4–8	NH_4_OH 0.0036–0.1	GLY 0.33–1	SCD	260–1025 °C	- GLY/TMOS molar ratio above 1.1 resulted in cracked samples, while 0.83 was the best ratio to obtain monoliths; - Larger pore radii were obtained when the post-treatment temperature increases from 260 to 650 °C.	[18] (1997)
TEOS 1	EtOH 40	2	NH_4_OH 0.0005	PEG Up to 10 mol%	APD or vacuum	-	- PEG allows a controlled texture; - The presence of PEG in SiO_2_ sols led to an increase in the particle size and then the formation of secondary particles with ring-like structures with short-order.	[11] (1998)
TMOS 1	MeOH 12	4	NH_4_OH 0.0036	GLY 0.2–0.8 Other: DMF, FA, Oxalic acid	SCD	-	- GLY leads to an increase in the specific surface area; - GLY is a suitable DCCA to produce monolithic aerogels.	[19] (1999)
TEOS 1	-	33	HNO_3_ 0.02 KOH 0.035	PEG 2.5–10.2 mg/mL of sol.	SCD with CO_2_	-	- With the increase of PEG concentration in the precursor system, the specific surface area decreased due to an increase in pore size; - Small concentrations of PEG increase the mechanical strength of the solid matrix.	[16] (2001)
TMOS/MTMS 1/0.7	MeOH 12	4	NH_4_OH 0.0036	GLY 0–0.2	SCD	-	- The lowest density and pores shrinkage were obtained for a GLY/TMOS molar ratio of 0.025.	[12] (2003)
TMOS 11 mL	-	See catalyst	Acid (acetic + citric) Aqueous solution pH = 5	PEG 2.45 g GLY solution	APD	550 °C	- PEG, together with citric acid, showed a control in the particle aggregation and internal structure; - Immersing the wet gel in a glycerol solution allows obtaining a crack free monolith.	[20] (2008)
Sodium Silicate 144 mL	-	525 mL	NH_4_OH pH = 4	GLY 3–5 wt%	APD	-	- Addition of GLY gives a more homogeneous microstructure; - GLY retards surface modification and solvent exchange; - The aerogel obtained with GLY maintained a relatively low bulk density compared with the aerogels aged in mixed ethanol/TEOS solution.	[13] (2008)
TEOS 0.5–1 mL/min	-	See catalyst	HCl 1.7 N	PEG 0.01–100 mmol/L	APD	600 °C	- Depending on the combination of molecular weight and concentration of the PEG solution, microporous and mesoporous silica materials can be obtained; - Texture of the produced silica is strongly correlated with polymer solution rheology.	[14] (2014)
MTMS 1	MeOH 35	See catalyst	Oxalic acid 4 NH_4_OH 4	PEG 0.01 Other: BTMSH and ODS	SCD with CO_2_	-	- PEG provides some uniformity to the porous network; - The addition of PEG leads to samples with lower densities, thermal conductivities and modulus.	[15] (2015)

^a^ Where not specified, the presented compounds quantities are the molar ratios; ^b^ TMOS—Tetramethylorthosilicate; MTMS—Methyltrimethoxysilane; TEOS—Tetraethylorthosilicate; MeOH—Methanol; EtOH—Ethanol; GLY—Glycerol; DMF—*N*,*N*-Dimethylformamide; FA—Formamide; PVA—Poly-vinyl alcohol; PEG—Poly(ethylene glycol); PAA—Polyacrylic acid; PEI—Polyethylene imine; BTMSH—bis(trimethoxysilyl) hexane; ODS—trimethoxy(octadecyl) silane; ^c^ SCD—Supercritical fluids drying; APD—Ambient pressure drying; ^d^ DCCA—drying control chemical additives.

**Table 2:** Samples identification and the molar ratios used for each aerogel-like material, as well as the corresponding densities and porosities.

Sample	MTMS:CH_3_OH:Acidic Water:Basic Water:Additive (Molar Ratio)	Bulk Density ^a^ (kg/m^3^)	Skeletal Density ^b^ (kg/m^3^)	Porosity (%)
S-0	1:35:4:4:0	79.9 ± 5.8	1223.5 ± 140.1	93.4
S-GLY-0.025	1:35:4:4:0.025	76.1 ± 3.3	1120.2 ± 59.7	93.2
S-GLY-0.05	1:35:4:4:0.05	79.9 ± 5.6	831.5 ± 36.0	90.4
S-GLY-0.075	1:35:4:4:0.075	83.4 ± 5.4	852.3 ± 21.3	90.2
S-GLY-0.1	1:35:4:4:0.1	84.2 ± 4.1	723.0 ± 8.0	88.4
S-PEG-0.025	1:35:4:4:0.025	72.7 ± 2.3	1543.3 ± 31.7	95.3
S-PEG-0.05	1:35:4:4:0.05	89.1 ± 6.7	1303.6 ± 79.2	93.2
S-PEG-0.075	1:35:4:4:0.075	89.4 ± 8.0	1319.2 ± 52.8	93.2
S-PEG-0.1	1:35:4:4:0.1	97.9 ± 11.9	1333.3 ± 265.7	92.7
S-PEG-0.025_W	1:35:4:4:0.025	46.1 ± 3.8	1684.8 ± 105.9	97.3

^a^ Uncertainties were calculated for a 95% confidence level; ^b^ Uncertainties were defined by the S.D.

**Table 3:** Specific surface area, pore volume and size of the aerogel-like materials with and without additives.

Sample	BET Specific Surface Area ^a^ (m^2^/g)	BJH-Desorption Pore Volume (cm^3^/g)	BJH-Desorption Aver. Pore Size ^b^ (Å)	Calculated Pore Volume ^c^ (cm^3^/g)	Calculated Pore Size ^d^ (nm)
S-0	400.3 ± 10.5	0.418	30.3	11.7 ± 1.0	116.8 ± 6.9
S-GLY-0.025	408.2 ± 7.2	0.417	28.2	12.3 ± 0.6	120.0 ± 3.9
S-GLY-0.1	347.0 ± 5.7	0.349	28.8	10.5 ± 0.6	121.0 ±4.8
S-PEG-0.025	374.5 ± 6.9	0.366	28.5	13.1 ± 0.4	140.0 ± 2.2
S-PEG-0.1	8.74 ± 0.14	0.015	39.4	9.5 ± 1.4	4331.6 ± 567.3
S-PEG-0.025_W	421.0 ± 8.8	0.574	37.3	21.1 ± 1.8	200.46 ± 13.2

^a^ Uncertainties were defined by the S.D.; ^b^ Detection interval: 10–1000 Å; ^c^ Pore volume = [(1/bulk density) − (1/skeletal density)]; ^d^ Pore size = (4*V*_P_)/*S*_BET_.

**Table 4:** Contact angles and thermal conductivities for the synthesized aerogel-like materials.

Sample	Contact Angle ^a^ (°)	Thermal Conductivity ^a^ (mW·m^−1^·K^−1^)
S-0	141.3 ± 1.7	38.65 ± 0.22
S-GLY-0.025	136.8 ± 0.9	38.28 ± 0.21
S-GLY-0.1	129.3 ± 3.1	39.98 ± 0.03
S-PEG-0.025	147.1 ± 6.0	39.01 ± 0.21
S-PEG-0.075 ^b^	134.5 ± 4.9	44.73 ± 0.26
S-PEG-0.025_W	146.6 ± 4.7	35.22 ± 0.25

^a^ Uncertainties were calculated for a 95% confidence level; ^b^ The sample S-PEG-0.1 was not monolithic to carry out the measurements.
